# Behavioral genetics and taste

**DOI:** 10.1186/1471-2202-8-S3-S3

**Published:** 2007-09-18

**Authors:** John D Boughter, Alexander A Bachmanov

**Affiliations:** 1 Department of Anatomy & Neurobiology, University of Tennessee Health Science Center, Memphis, TN 38163, USA; 2 Monell Chemical Senses Center, 3500 Market Street, Philadelphia, PA 19104-3308, USA

## Abstract

This review focuses on behavioral genetic studies of sweet, umami, bitter and salt taste responses in mammals. Studies involving mouse inbred strain comparisons and genetic analyses, and their impact on elucidation of taste receptors and transduction mechanisms are discussed. Finally, the effect of genetic variation in taste responsiveness on complex traits such as drug intake is considered. Recent advances in development of genomic resources make behavioral genetics a powerful approach for understanding mechanisms of taste.

## Introduction

The chemical senses, gustation and olfaction, have provided a candidate media for investigation of the effects of major genes on behavior, owing to considerable natural variation for both senses among individuals or strains of mice and rats. Genetic analysis of behavioural responses to bitter and sweet taste stimuli in mice facilitated the recent discovery of receptor gene families for bitter, sweet and amino acid taste (for recent reviews, see [1-4]). The function and specificity of taste receptors continues to be elucidated based on not only *in vitro *expression assays and transgenic approaches, but also based on studies of gene variation and evolution (e.g. [5-8]).

Taste, beyond its purely sensory function, is also inextricably linked to a larger set of behaviors. The gustatory system is anatomically located at the beginning of the alimentary canal, and as such is a key player in homeostatic systems dealing with nutrient and fluid intake. Taste and feeding are linked in terms of reciprocal connectivity between brain centers (e.g. [9,10]). The consumption of sweeteners, for example, often involves a complex integration of the peripheral sensory system, the central nervous system and post-ingestive events. Sweet taste detection in mammals has been shown to be heavily modulated by post-ingestive feedback, including neural or hormonal factors (e.g. [11-13]). This feedback extends to the level of the taste receptor cells and afferent nerves [14,15]. Nutritive sweeteners provide post-ingestive stimulation [16], probably due to their calories. These interactive mechanisms of consummatory responses to sweeteners imply that ingestion is a complex behavior, and is likely to be determined by multiple genes.

Although studies of non-mammalian organisms such as fruit flies hold tremendous potential for elucidating genetic basis of taste behavior and physiology [17,18] this review will focus on studies of taste genetics in mammals, primarily in mice, and to a lesser extent in rats and humans. In particular, we will concentrate on the characterization of phenotypic diversity among inbred strains of mice, as this naturally-occurring variation has proved crucial for genetic analysis and the eventual identification of G-protein coupled taste receptors. Hence, the review is arranged by taste quality, including those characterized by humans as tasting sweet, umami (savory), bitter and salty. Although candidate sour taste receptor molecules have been recently identified [19,20], less progress has been made regarding genetic approaches to sour (acid) taste, and this quality is not discussed here.

### Measuring taste behavior

Taste perception involves several aspects, such as intensity, quality and hedonic value of the taste sensation. In humans, these aspects of taste perception are usually assessed using verbal information, e.g., by plotting sensation intensity on a scale with verbal descriptors or by reporting a difference between samples. Early studies of human genetic variation of taste sensitivity concentrated on either responses to a single concentration of stimulus (i.e., tasters vs. non-tasters of phenylthiocarbamide (PTC)) or measurement of absolute thresholds [21]. A more expansive view of taste ability, and therefore phenotypic differences, may be gained from analyses of the perceived taste intensity [22,23].

Assessment of taste perception in non-human animals relies on recording of behavior elicited by taste stimuli using a number of different techniques [24]. Each of these techniques characterizes some, but not all aspects of taste perception. For example, hedonics of taste sensation can be assessed based on consummatory responses in naive animals, but assessing taste quality involves conditioning of animals. Many of these techniques have been adapted for use in mice and can be used in genetic experiments (Table 1).

**Table 1:** Methods to characterize taste in non-human animals

**Information**	**Method**			
	
	**Long-term two-bottle preference**	**Brief-access licking responses**	**CTA**	**Electrophysiological recording of taste-evoked activity in gustatory nerves**
Sensitivity (threshold)	+/- ^a^	+/- ^a^	+	+
Intensity	+	+	-	+
Hedonic (pleasant or unpleasant) properties	+	+	-	-
Taste quality	-	-	+	+/-^b^
Specificity	Can be influenced by post-ingestive effects	Minimizes postingestive effects; can be influenced by water restriction	Can be influenced by non-taste sensory properties (e.g., odor), learning ability or sensitivity to an unconditioned stimulus	Directly assesses peripheral neural response

+, test is primarily designed to assess a particular aspect of taste perception (e.g., intensity or quality); +/-, limited information regarding the aspect of taste perception can be derived from test results; -, test is not informative regarding the aspect of taste perception. ^a ^Response thresholds of naive (not conditioned) animals are often higher that taste recognition thresholds in conditioned animals. ^b ^Taste quality can be assessed using analysis of single-fiber activity, but not using integrated whole-nerve recordings. All types of tests have been used in genetic experiments, but two-bottle preference tests have the highest throughput of phenotyping.

Traditionally, behavior genetic studies of taste in the mouse have been conducted using two-bottle intake tests, e.g. [25-28]. These tests simply measure the consumption of a taste stimulus over a 24- or 48-h period, relative to the consumption of water. Sweet-tasting stimuli are generally preferred to water, whereas bitter-tasting stimuli are avoided. Such tests, while amenable for testing large cohorts of mice required for genetic analysis, do not easily allow for the dissociation of sensory ability from post-ingestive factors such as satiety or toxicity. Brief-access tests minimize post-ingestive cues by restricting trial lengths to short durations (< 30 s). The dependent measure is number of licks taken from a particular stimulus, typically expressed as a ratio (licks to stimulus/licks to water). Such tests have been effectively used for high-throughput screening of taste function in mouse populations, especially for compounds with an aversive taste [29,30].

Other tests of taste function in the mouse include measurements of threshold detection or the ability to discriminate between two stimuli [31,32]. Conditioned taste aversion (CTA) is a commonly used paradigm to estimate perceptual similarity or dissimilarity between stimuli. The presentation of a novel taste stimulus is paired with a stimulus that produces temporary gastric distress, usually an intraperitoneal dose of LiCl. After pairing, the animal will avoid consumption of the conditioned taste stimulus, and this CTA will generalize to stimuli with similar perceptual qualities.

Finally, electrophysiological recordings of activity in the afferent gustatory nerves often corroborate behavioral taste tests in non-human animals. The major gustatory nerves are the chorda tympani (CT) branch of the facial (VII) nerve and the glossopharyngeal (IX) nerve. The CT predominantly innervates taste buds on the anterior tongue, whereas the IXth nerve innervates taste buds contained in trenches on the posterior tongue. Studies of gustatory nerve activity help to elucidate whether genetic effects on taste perception have peripheral or central origin.

### Genetic analysis of taste behavior

In genetic terms, the taste response of an individual represents a phenotype, i.e., an observable property of an organism produced by genetic and environmental factors. A typical goal of genetic studies is to characterize the genetic factors contributing to the phenotype. This is usually achieved by analyzing populations of related individuals, such as twins or families in humans, or crosses between inbred strains of mice or rats. An initial goal of the genetic analysis is to establish whether the phenotypical variation among individuals has a genetic component (i.e., is heritable). Once heritability is confirmed, chromosomal localization of genes responsible for the heritable component of the phenotype can be found using linkage analysis. The linkage analysis involves assessment of association between genetic markers distributed throughout the genome and the phenotype. Some linkage studies in rodents use recombinant inbred strains, which are produced by intercrossing two parental inbred strains, obtaining the second generation of hybrids (F_2_), and subsequent inbreeding (brother × sister mating) of multiple strains. After a genetic locus affecting a phenotype is mapped to a particular chromosome (Chr), subsequent studies can identify the molecular nature of the corresponding gene. Because gene identification is based primarily on the chromosomal position of the gene, this approach is called positional cloning.

## Sweet and umami taste

### Genetic variation in sweet taste responsiveness

Taste sensitivity to sweeteners varies among individual humans, and this variation may be determined genetically [33-42]. Prominent genetic differences in taste responses to sweeteners also exist among inbred strains of mice. These differences were shown using different experimental techniques and a variety of sweeteners (such as sucrose, glucose, dulcin, saccharin, acesulfame, glycine, D-phenylalanine and L-glutamine). Mice from different strains vary in taste responses to sweeteners assessed using long-term preference tests [26,27,43-53], single-bottle tests [54], brief-access tests based on lick recording [55,56], taste detection thresholds [31], CTA generalization [57], and responses of gustatory nerves [58-60]. These studies have shown that responses to many of these sweeteners (e.g., sucrose, glucose, dulcin, saccharin and acesulfame) closely correlate among mouse strains, suggesting a common genetic basis for sweet taste. However, responses to some sweet-tasting amino acids display somewhat different patterns of strain differences. Strain differences in consummatory responses to sweeteners have also been reported for rats [61-63] and hamsters [64].

A few mouse strains with large differences in sweet taste responses were used to produce crosses for analyses of genetic and physiological mechanisms underlying behavioral responses to sweeteners. The most detailed physiological analysis was conducted using mice from the C57BL/6 (B6) strain with high sweetener preferences and mice from the 129 strains with low sweetener preferences. Compared with 129 mice, B6 mice had higher preferences for a large number of sweeteners, including sugars (sucrose and maltose), sweet-tasting amino acids (glycine, D-phenylalanine, D-tryptophan, L-proline and L-glutamine), and several but not all non-caloric sweeteners (saccharin, acesulfame, dulcin, sucralose and SC-45647) [46,50-52,65]. This phenotypic difference is specific to sweet-taste processing, and is not due to a generalized difference in taste responsiveness or differences in appetite [66,67].

Differences between B6 and 129 mice in preference for a sweet-tasting amino acid glycine [65] appear to depend on mechanisms distinct from those affecting responses to many other sweeteners. Both B6 and 129 mice generalized a CTA between glycine and several other sweeteners, demonstrating that they perceive the sucrose-like taste of glycine. Thus, the lack of a strong glycine preference by 129 mice cannot be explained by their inability to perceive its sweetness [68]. Despite differences in glycine intakes and preferences, CT responses to glycine are similar in mice from both strains [58]. Neither behavioral nor neural responses to glycine are influenced by the *Tas1r3 *genotype [31,69], suggesting that variation in taste responses to glycine depends on other genes.

Some genetic analyses of sweetener consumption by mice yielded evidence that it is influenced by a single locus, named *Sac *(saccharin preference) [46,50,70,71], whereas other experiments indicated that more than one gene is involved [47,50,51,72,73]. The apparent discrepancy on whether single-gene or multi-gene model better describes genetic variation in sweetener preferences is likely due to use of different progenitor strains and types of mapping panels, different sweetener solutions tested, and different quantitative analyses used in these studies.

### The saccharin preference (*Sac*) locus and the *Tas1r3 *gene

Using long-term two-bottle tests, Fuller [70] demonstrated that differences in saccharin preferences between the B6 and DBA/2J inbred strains largely depend on a single locus, *Sac*, with a dominant *Sac*^*b *^allele present in the B6 strain associated with higher saccharin preference, and a recessive *Sac*^*d *^allele present in the DBA/2J strain associated with lower saccharin preference. Subsequent studies confirmed this finding in the BXD recombinant inbred strains, and in crosses between the B6 and DBA/2 or between the B6 and 129 strains [46,50,71,72,74,75]. In addition to sweetener preferences, the *Sac *genotype influences the afferent responses of gustatory nerves to sweeteners [75,76], which indicated that the *Sac *gene is involved in peripheral taste transduction and may encode a sweet taste receptor.

**Figure 1:**
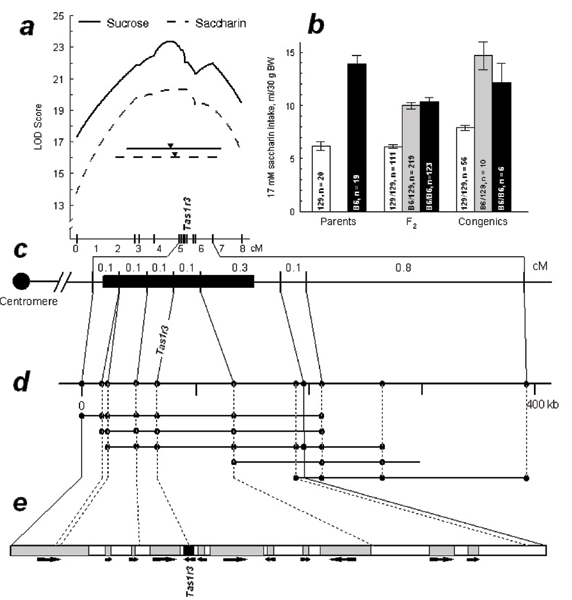
**Positional identification of the *Sac *(saccharin preference) locus.***a*. Linkage map of mouse distal Chr 4 based on data from the B6 × 129 F_2 _intercross. The X axis shows distances between markers in recombination units (cM). The Y axis shows the logarithm of the odds ratio (LOD) scores for sucrose and saccharin consumption. The LOD score peaks (indicated by black triangles) and confidence intervals (solid horizontal line for sucrose, 4.5 cM, and dotted horizontal line for saccharin, 5.3 cM) define the genomic region of the *Sac *locus. *b*. Average daily 17 mM saccharin consumption by mice from parental 129 and B6 strains (left), B6 × 129 F_2 _hybrids (center), and congenic 129.B6-*Sac *mice (right) in 96-hr two-bottle tests with water (means ± SE). *Tas1r3 *genotypes of the F_2 _and congenic mice and mouse numbers are indicated on the bars. Differences between parental strains and among the F_2 _and congenic genotypes were significant (*p *< 0.0001, ANOVA). F_2 _and congenic B6 homozygotes and heterozygotes for *Tas1r3 *did not differ from each other, and had higher saccharin intakes compared with 129 homozygotes (*p *< 0.0001, post hoc tests). *c*. Linkage map of the *Sac*-containing region defined based on the size of the donor fragment in the 129.B6-*Sac *congenic strain (black box). Distances between markers were estimated based on the B6 × 129 F_2 _intercross (see panel *a*). *d*. A contig of bacterial artificial chromosome (BAC) clones and physical map of the *Sac *region. BAC clones are represented by horizontal lines. Dots indicate marker content of the BAC clones. *e*. Genes within the *Sac*-containing interval. Filled areas indicate predicted genes. Arrows indicate the predicted direction of transcription. Figure reproduced with permission from [253].

The *Sac *locus has been mapped to the subtelomeric region of mouse Chr 4 [50,72,74-76] (see also Figure 1). A positional cloning study at the Monell Chemical Senses Center showed that the *Sac *locus corresponds to the *Tas1r3 *gene [77,78]. This study involved a high-resolution linkage analysis of a B6 × 129 F_2 _intercross, the marker-assisted selection of a 129.B6-*Sac *congenic strain, physical mapping that involved construction of a contig of bacterial artificial chromosome (BAC) clones, BAC sequencing, and sequence analysis of candidate genes. One of the genes within the critical interval of the *Sac *locus was a G protein-coupled receptor gene, *Tas1r3 *(taste receptor, type 1, member 3). Based on the effects of the *Sac *genotype on peripheral sweet taste responsiveness [75,76], and on involvement of a G protein-coupled mechanism in sweet taste transduction [79], *Tas1r3 *was selected as the most likely candidate for the *Sac *locus (Figure 1).

The *Tas1r3 *gene contains 6 coding exons and is translated into an 858-amino acid protein, T1R3, with a predicted secondary structure that includes seven transmembrane domains and a large hydrophilic extracellular N-terminus. This structure is typical of the class C G protein-coupled receptor family, which includes the metabotropic glutamate and extracellular calcium-sensing receptors. T1R3 belongs to a small family of G protein coupled receptors, T1R, which also includes the T1R1 and T1R2 proteins. The three mouse T1R genes are located on distal Chr 4 in the order: *Tas1r2 *(70.0 cM, or 139 Mb, NCBI Build 36) – *Tas1r1 *(81.5 cM, or 151 Mb) – *Tas1r3 *(83.0 cM, or 155 Mb). The *Tas1r1 *and *Tas1r2 *genes were excluded as candidates for the *Sac *locus based on their more proximal chromosomal location [76,80,81]. All three T1R members are expressed in taste receptor cells [53,80-85]. When T1R3 is co-expressed in a heterologous system with T1R2, it functions as a sweet receptor [85-87], but T1R3 may also function as a low-affinity sugar receptor alone, probably as a homodimer [88]. *Tas1r3 *has a human ortholog, *TAS1R3*, residing in a region of conserved synteny in the short arm of human Chr 1 (1p36) [89].

If *Tas1r3 *is identical to *Sac*, substitution of *Tas1r3 *alleles must result in phenotypical changes attributed to the *Sac *locus. Introgression of the 194-kb chromosomal fragment containing the *Tas1r3 *allele from the high-sweetener preferring B6 strain onto the genetic background of the 129 strain fully rescued its low sweetener preference phenotype: sweetener intake of the congenic mice was as high as that of mice from the donor B6 strain [77] (Figure 1). Equivalent phenotype rescue results were obtained in a transgenic experiment [85]. These data demonstrate that substitution of *Tas1r3 *alleles results in behavioral changes attributed to the *Sac *locus and therefore provides a proof that *Tas1r3 *is identical to *Sac*, and that the T1R3 receptor responds to sweeteners. Further evidence for identity of the *Sac *locus and *Tas1r3 *gene was obtained in studies of mice with targeted mutations of the *Tas1r3 *gene, which were found to be deficient in taste responses to sweeteners [88,90]

Identity of *Sac *and *Tas1r3 *implies that there must be *Tas1r3 *polymorphisms, resulting in variation of sweet taste responses attributed to allelic variants of the *Sac *locus. Although several candidate functional polymorphisms were proposed in studies that identified the *Tas1r3 *gene [80,81,83-85], these studies lacked a proper quantitative analyses of gene-phenotype associations.

**Figure 2:**
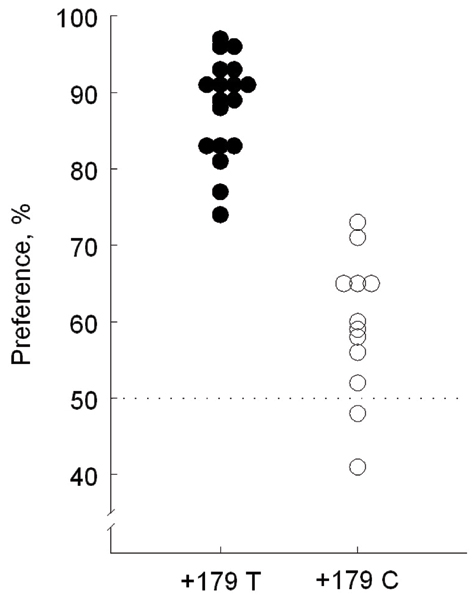
**Preference for 1.6 mM saccharin by mice from inbred strains with different *Tas1r3 *genotypes at the T/C variant site at nucleotide position +179 (relative to the first nucleotide in the ATG start codon of the *Tas1r3 *gene). **This polymorphism results in amino acid substitution of isoleucine to threonine at position 60 (I60T), in the extracellular N-terminus of the predicted T1R3 protein. Closed circles denote means for C57BL/6, C57L/J, CAST/Ei, CE/J, FVB/NJ, I/LnJ, IS/Cam, KK/HlJ, NOD/LtJ, NZB/BlNJ, P/J, RBF/DnJ, SEA/GnJ, SJL/J, SM/J, SPRET/Ei, ST/bJ and SWR/J strains with +179 T genotype. Mice from these strains strongly preferred saccharin (average preference score 88 ± 2%, Mean ± SE; n = 18). Open circles show means for 129P3/J, A/J, AKR/J, BALB/cByJ, BUB/BnJ, C3H/HeJ, CBA/J, DBA/2J, LP/J, PL/J, RF/J and RIIIS/J strains with +179 C genotype. Mice from these strains were indifferent to or only weakly preferred saccharin (average preference score 59 ± 3%, n = 12; p = 0.00000000012, t-test). Despite the strong phenotypical effect of the *Tas1r3 *genotype, there is also substantial variation in saccharin preference within each genotype group. As a result, *Tas1r3 *genotype explains only 78% of genetic variation in saccharin preferences among the inbred strains; the remaining 22% of genetic variance is attributed to the effect of other genes. Adapted with permission from [53].

Reed et al [53] conducted a comprehensive quantitative analysis of the *Tas1r3 *sequence variants associated with saccharin preference using 30 genealogically diverse inbred mouse strains. Genomic sequences including *Tas1r3 *exons, introns, upstream and downstream regions were examined, so that polymorphisms affecting amino acid composition or potential regulatory regions could be detected. The strongest association with saccharin preference was found for a haplotype including three sites: nucleotide (nt) -791 (3 bp insertion/deletion), nt +135 (Ser45Ser), and nt +179 (Ile60Thr) (Figure 2). Lack of differences in the *Tas1r3 *gene expression in the taste tissues of mice with different *Tas1r3 *haplotypes suggested that the polymorphisms that do not change amino acid sequence of the T1R3 protein (nt -791 and nt +135) are unlikely to affect receptor function. Therefore, the amino acid substitution of isoleucine to threonine at position 60 (Ile60Thr) was predicted to be a functional polymorphism [53]. This prediction was subsequently confirmed in an *in vitro *study showing that a corresponding site-directed mutation changes binding affinity of the T1R3 protein to several sweeteners [91].

### Ligand specificity of the T1R3 receptor

*In vitro *studies of T1R proteins have indicated that they function as broad-spectrum sweet and umami receptors (Table 2) [85,87,92]. Several aspects of these *in vitro *studies emphasize importance of an *in vivo *approach to characterize ligand-receptor interactions. First, discrepancies between results obtained using different expression systems leave open a question of whether responsiveness or unresponsiveness to a particular sweetener reflects *in vivo *sensitivity of the receptor, or is an artifact of the *in vitro *system. Second, responses of the heterologously expressed mouse receptors to amino acids are not always consistent with mouse behavioral responses to these stimuli. For example, sweet L-proline and L-threonine did not activate the T1R2 and T1R3 combination, but instead activated the T1R1 and T1R3 combination, which also responded to some umami-tasting and bitter (e.g., L-phenylalanine) compounds [87].

**Table 2:** Deorphanized taste receptors

	**Receptors**	
**Ligands**	**Human**	**Mouse**

Sugars, sweeteners, D-amino acids^1^	hT1R2 + hT1R3	mT1R2 + mT1R3
Umami stimuli and L-amino acids	hT1R1 + hT1R3	mT1R1 + mT1R3
Beta-glucopyranosides	hT2R16	
PROP	hT2R4^2^	mT2R8^2^
Structurally diverse set of bitter stimuli	hT2R14	
Strychnine	hT2R10	
Saccharin, acesulfame K	hT2R44, hT2R43	
PTC	hT2R38	
Denatonium	hT2R4, hT2R44	mT2R8
Cycloheximide		mT2R5

The ligand specificities have been determined predominantly by *in vitro *studies, but also through the use of knock-out or transgenic mice, as well as other approaches examining allelic variance. The sweet and amino acid receptors have broad ligand specificity. The T1R2 + T1R3 heterodimer responds to a broad array of sweet-tasting stimuli in both human and mouse [85–87]. T1R1 + T1R3 responds to umami stimuli in humans and to a wider range of stimuli including a variety of L-amino acids in mice [86, 87]. A T1R3 homodimer may respond to sugars at higher concentrations [88, 90]. In contrast, most of the bitter receptors have narrow ligand specificities [111, 112, 125, 250, 251]. However, hT2R14 has been shown to respond to a broad group of bitter compounds, including plant neurotoxins [252].

^1^Excepting D-glutamate and D-aspartate.

^2^Only high concentrations of PROP were found to activate this receptor *in vitro*.

The *in vivo *approach to characterize ligand specificity of the T1R3 receptor is based on an assumption that if a response to a compound is affected by *Tas1r3 *genotype, then this compound activates a receptor involving T1R3. Several earlier genetic mapping studies have shown that in addition to saccharin preferences, the *Sac *locus also affects consumption of sucrose, acesulfame [46,50,74,75], and ethanol, which has a sweet taste component [93], and also chorda tympani responses to saccharin and sucrose [75,76]. Inoue et al. [69] examined behavioral and neural responses to a larger set of sweeteners in the F_2 _hybrids between the B6 and 129 strains. They found that the *Tas1r3 *genotype affected consumption of the sweeteners sucrose, saccharin and D-phenylalanine, but not glycine. For CT responses, significant linkages to *Tas1r3 *were found for the sweeteners sucrose, saccharin, D-phenylalanine, D-tryptophan and SC-45647, but not glycine, L-proline, L-alanine or L-glutamine. No linkages to the *Tas1r3 *chromosomal region were detected for behavioral or neural responses to non-sweet quinine, citric acid, HCl, NaCl, KCl, monosodium glutamate (MSG), inosine 5'-monophosphate (IMP) or ammonium glutamate. Thus, allelic variation of the *Tas1r3 *gene affects taste responses to many but not all sweeteners, suggesting that a wide variety of sweeteners can activate a receptor involving T1R3. Lack of the effect of the *Tas1r3 *genotype on taste responses to some sweeteners, such as glycine, can be explained by several possible mechanisms: (i) sweetener binding to the T1R3 receptor at a site that is not affected by the polymorphic variants; (ii) binding to the T1R2 receptor; (iii) existence of another sweet taste receptor binding these sweeteners. These mechanisms can be examined using mice with targeted mutations of the *Tas1r *genes, but there are currently no data on taste responses to glycine in *Tas1r *knockout mice.

### Other genes involved in sweet taste responses

Multigenic inheritance of sweetener preferences was shown in a number of studies [47,50,51,72,73]. Accordingly, several lines of evidence indicated that allelic variation of the mouse *Tas1r3 *locus does not account for all the genetically determined differences in sweetener preferences. Analysis of multiple inbred mouse strains has shown that the *Tas1r3 *genotype explains only 78% of genetic variation in saccharin preference [53] (Figure 2). In the B6 × 129 F_2 _cross, the *Tas1r3 *genotype explained 64 – 96% of genetic variation in preference scores for different sweeteners, 10 – 35% of genetic variation in sweetener intakes, and 37 – 92% of genetic variation in CT responses to sweeteners [69,75]. Responses to sweeteners in brief-access tests differ among mouse strains but do not seem to be associated with *Tas1r3 *alleles [55]. Thus, a substantial part of the genetic variation in taste responses to sweeteners among mouse strains is attributed to loci other than *Tas1r3*. Taste responses to glycine provide a remarkable example: although there are substantial differences among mouse strains in responses to glycine [50,65], this variation is not attributed to the *Tas1r3 *genotypes [31,69]. Consistent with the mouse work suggesting effects of genes other than *Tas1r3*, variation in saccharin preferences in rats is not associated with sequence variants of the rat *Tas1r3 *gene [94], and therefore it must be attributed to the effects of other genes (see also [95]).

Because a sweet taste receptor appears to be a heterodimer of T1R2 and T1R3 proteins, sequence variants of the *Tas1r2 *gene potentially can also result in variation in sweet taste responses. There is no evidence that *Tas1r2 *variation contributes to mouse strain differences in responses to sweeteners. However, sequence variation among the *Tas1r2 *orthologs underlies some species differences in sweet taste perception. Inability of rodents to perceive sweetness of several compounds that taste sweet to humans is attributed to variation between human and rodent orthologs of *Tas1r2 *[87,96]. Lack of preference for sugars and some other sweeteners in cats (Felidae) [97,98] is due to pseudogenization of the *Tas1r2 *gene, and thus inability to produce a T1R2 + T1R3 heterodimeric sweet taste receptor [8].

One of the genetic loci affecting sweet taste responses is *dpa *(D-phenylalanine aversion), which affects ability of mice to generalize a CTA between D-phenylalanine and sucrose, inferring that *dpa *affects ability to detect sweetness of D-phenylalanine. The *dpa *locus also affects responses of sucrose-sensitive fibers of the CT nerve to D-phenylalanine. B6 mice carry a dominant allele of *dpa *that determines an ability to recognize the sweetness of D-phenylalanine, whereas BALB/c mice carry a recessive *dpa *allele conferring inability to detect D-phenylalanine sweetness. The *dpa *locus was mapped to proximal Chr 4, a region distinct from the subtelomeric Chr 4 harboring the *Tas1r *genes [99-102]. It was suggested that the *dpa *locus can also affect responses to sweeteners in two-bottle tests [51]. Consistent with this, a locus on proximal Chr 4, in the *dpa *region, was found to be suggestively linked to consumption of, and CT responses to, sucrose [75]. An epistatic interaction between effects on sucrose intake of this locus and the *Tas1r3 *locus suggests that these two loci may encode interacting components of sweet taste transduction [75].

In summary, these data show that sweetener preference has a complex genetic determination. In addition to the *Tas1r3 *gene, other genetic loci play the role in genetic variation of taste responses to sweeteners. These other genes may encode additional peripheral taste transduction elements, including novel taste receptors. Alternatively, they may be involved in central mechanisms of ingestive behavioral responses or feedback mechanisms of modulation of sweet taste responses.

### Umami taste genetics

Umami is a taste quality exemplified by taste of MSG and evoked also by some amino acids and purine 5'-nucleotides. There is a strong evidence that a heterodimer of T1R1 and T1R3 proteins functions as an umami taste receptor in humans and is a more broadly tuned in rodents to respond to L-amino acids (Table 2) [87,88,90,92]. A splice variant of a metabotropic glutamate receptor, mGluR4, was also proposed as a taste receptor for glutamate [103].

Little is known about genetic variation in umami taste responses. Humans appear to differ in perception of glutamate taste [104], but it is not known whether there is a genetic basis for this. A comparison of the CT nerve responses in three inbred mouse strains has shown differences in a synergistic effect between MSG and 5'-guanylate [105].

In the long-term two-bottle preference tests, mice from the B6 strain consumed more MSG and IMP than did mice from the 129 strain [106]. The strain difference in MSG consumption was in the opposite direction to the strain difference in NaCl consumption [66,107]. Although the B6 mice have higher avidity for both MSG and sweeteners than do the 129 mice [65,66,106], there is no correlation between preferences for these solutions in the F_2 _hybrids derived from these two strains [106]. Thus, differences in MSG consumption between B6 and 129 mice are not related to the strain differences in salty or sweet taste responsiveness.

The role of the afferent gustatory input in these strain differences was examined by measuring integrated responses of the CT and IX nerves to umami taste stimuli. In the CT, responses to MSG and monoammonium L-glutamate were similar in B6 and 129 mice, but responses to IMP and guanosine-5'-monophosphate were lower in B6 than in 129 mice. Responses to umami stimuli in the IX nerve did not differ between the B6 and 129 strains [108]. Thus, the increased ingestive responses to the umami stimuli in B6 mice are accompanied by either unchanged or decreased neural responses to these stimuli. Lack of support for the role of the gustatory nerves in the enhanced consumption of MSG and IMP by B6 mice suggests that it is due to some other factors. A prior exposure to MSG affects subsequent MSG consumption [106], suggesting that it can be modulated by postingestive effects. The strain differences in gustatory neural responses to nucleotides but not glutamate [108] suggest that these compounds may activate distinct taste transduction mechanisms.

The T1R3 protein is involved in transduction of both sweet and umami tastes, and a disruption of the *Tas1r3 *gene diminishes behavioral or neural responses to umami taste stimuli [86-88,90]. Hence, variation of the *Tas1r3 *gene might affect umami taste responses. However, an analysis of the F_2 _hybrids between the B6 and 129 inbred mouse strains has shown that the *Tas1r3 *allelic variants do not affect behavioral or neural taste responses to umami stimuli [69]. Thus, although the T1R3 receptor is involved in transduction of umami taste, the B6/129 sequence variants affecting its sensitivity to sweeteners do not affect its sensitivity to umami compounds.

## Bitter taste

### Bitter taste receptor genes

A large and diverse array of molecules evoke the sensation of bitterness, and the ability to detect and avoid these stimuli is assumed to have evolved as a mechanism to prevent ingestion of toxic foods [109]. Indeed, a strong correlation exists in the animal kingdom between bitter sensitivity and tolerance to toxic compounds [110]. A *Tas2r *family of G-protein coupled receptor genes linked to bitter taste sensitivity was identified in 2000 by laboratories led by Nicholas Ryba, Charles Zuker [111,112] and Linda Buck [113]. The T2R receptors are related to class A GPCRs and are characterized by a short N terminus with a potential transmembrane ligand-binding domain. To date, about 28 human and 36 mouse intact bitter taste receptor genes have been identified, although only a few have been de-orphanized (Table 2) [114].

### The PTC polymorphism in humans and mice

The first example of a genetic basis to taste ability involved the serendipitous discovery of human differences in sensitivity to the bitter-tasting compound PTC [115]. Population surveys indicated a dimorphism in taste sensitivity for this substance, with individuals falling into two categories: "tasters" and "nontasters" [116,117]. Pedigree analysis suggested phenotypic control by a single, autosomal locus [118,119] although more recent studies postulated that PTC inheritance was better described by a polygenic model [120,121]. Linkage studies in humans supported associations with several chromosomal loci, including the KEL blood group antigen on Chr 7q (e.g. [122,123]). Dennis Drayna and colleagues [124,125] have shown that this locus corresponds to a polymorphic bitter taste receptor gene *TAS2R38* responsible for up to 85% of the variation in PTC sensitivity among human subjects. A secondary QTL on Chr 16p also has an effect on PTC aversion, although the nature of this gene is not known [124].

**Figure 3:**
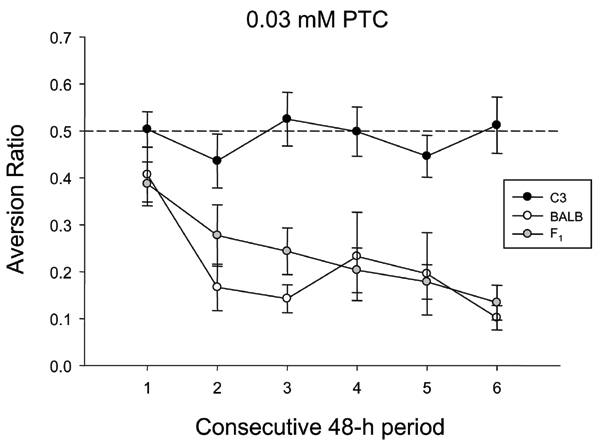
**Aversion to PTC in taster mice develops over several days**. Preference ratios (mean ± SE) for BALB/cBy (BALB), C3HeB/Fe (C3), and BALB × C3 F_1 _(n = 10/strain) to 0.03 mM PTC over six consecutive 48-h tests. The dotted line represents a preference score of 0.5, which indicates equivalent consumption from solution and water tubes. Preference ratios for BALB and F_1 _mice decreased following the initial test period; by the final test period these mice were strongly avoiding PTC. C3 mice remained indifferent to the taste of PTC across the entire test period. Strain differences in "developed aversion" to PTC have a genetic basis, with complete dominance of the avoider phenotype. However, the avoidance phenotype does not necessarily reflect increased bitter taste sensitivity. Figure from Boughter (unpublished).

From the early discovery of human variation in PTC taste sensitivity, investigators proceeded to animal models of bitter taste genetics. Richter and Clisby [126] reported individual variation in PTC avoidance thresholds of rats, but the genetic basis for this was not pursued. Klein and DeFries [127] found that two-bottle intake of PTC varied in mice, with BALB/c mice significantly more sensitive to the bitter taste of PTC than other inbred strains, including C57BL/Ibg. A Mendelian cross (F_1_, F_2_, F_3_, B_1_, and B_2 _generations) between these two strains yielded segregation ratios consistent with phenotypic control by a single autosomal locus, with a dominant taster allele. It was later recognized that PTC avoidance by BALB/c mice generally develops across 10–12 consecutive days of testing [128] (Figure 3). PTC is extremely toxic to mice (oral LD_50 _10 mg/kg), comparable to the rodent poison strychnine (oral LD_50 _2 mg/kg). Confining stimulus access to shorter durations helps to minimize or eliminate post-ingestive consequences (e.g. [29,129-132]). A direct comparison of intake tests and brief-access procedures demonstrated that differential avoidance of PTC among inbred strains depended on the amount of stimulus consumed, and not necessarily on immediate orosensory or taste cues [133]. When injected intraperitoneally, PTC was nearly as effective as LiCl in serving as the unconditioned stimulus in a CTA experiment [134]. Strain differences in PTC avoidance that develop over time therefore likely reflect other variables in addition to sensory ability, such as sensitivity to its toxic effects, or differences in the speed of acquisition of a CTA.

### Sucrose octaacetate (SOA) and quinine aversions and bitter taste receptor genes

The genetic basis of differences in intake of bitter compounds other than PTC has been studied throughout the last several decades, including substantial contributions from the laboratories of Ian Lush and Glayde Whitney (see [135,136]). Inbred strains differ in sensitivity to the commonly-used bitter stimuli, quinine and SOA, assessed using two-bottle preference tests [66,137-140]. For SOA, the strain difference is particularly robust as it occurs over a 2–3 log-step concentration range. SOA aversion appeared to be a fairly unambiguous model of monogenic control: Aversion ratios among segregating generations of mice were consistent with variation at a single autosomal locus (*Soa*, sucrose octaacetate aversion), with the taster allele dominant over either a "demitaster" (partial sensitivity) or nontaster allele [138,141,142]. Results of both brief-access behavioral tests and peripheral taste nerve electrophysiology yielded concentration-by-concentration similarity with two-bottle data, confirming that the strain difference was gustatory in nature [132,140,143]. Relevant to this finding is the fact that SOA is a non-toxic, apparently non-odorous compound. *Soa *was subsequently mapped to distal mouse Chr 6 near proline-rich salivary protein genes *Prp2 *and *Prh1 *[136,144,145]. At least one other study has found evidence for polygenic control of SOA aversion [146], and interestingly, the aversion phenotype is subject to environmental modulation: unlike control tasters, taster mice raised on drinking water adulterated with a normally avoided concentration of SOA display only minimal avoidance of SOA in a two-bottle test with water [147].

**Figure 4:**
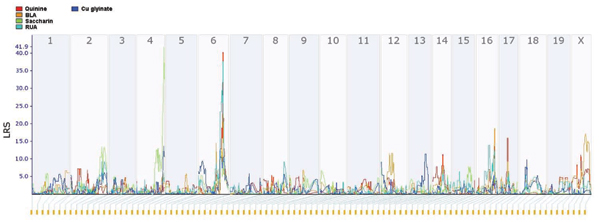
**Genome-wide interval mapping of taste responses to bitter and sweet compounds:** 0.1 – 0.4 mg/ml quinine, 0.3 mM beta-lactose acetate (BLA), 0.4 mM raffinose undecaacetate (RUA), 10 mM copper glycinate and 3.2 mM saccharin. LRS = Likelihood Ratio Score. The taste stimuli were tested in the two-bottle preference tests in the BXD recombinant inbred mouse strains. Phenotype data were previously published [46, 72, 151, 153] and are available at . Mapping was conducted using WebQTL software . The interval mapping illustrates previously published linkages for avoidance of all four bitter-tasting stimuli to Chr 6 and for saccharin preference to Chr 4 (*Sac/Tas1r3 *locus). A recent study of bitter taste in BXD mice using brief-access procedures demonstrates that the QTL on Chr 6 reflects orosensory processing, and not post-ingestive effects, of the bitter stimulus quinine [30].

**Figure 5:**
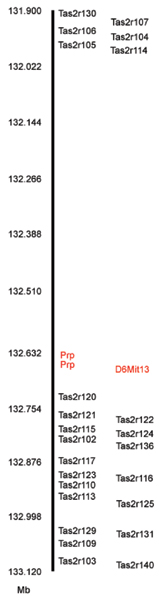
**A map of the cluster of the *Tas2r *bitter taste genes on distal mouse Chr 6.** Twenty-four intact *Tas2r *genes map to distal Chr 6 (black). The *Tas2rs *are found in two subclusters on either side of the polymorphic marker *D6Mit13 *(red) and two genes encoding proline-rich salivary proteins (*Prp2 *and *Prh1*; red). Map positions are in Mb (Build 33 assembly of the B6 genome). Figure copyright [30].

Unlike responses to SOA, responses to quinine in two-bottle tests were demonstrated to be under polygenic control in segregating crosses or recombinant inbred strains [148-150]. However, linkage studies positioned a locus with major effects on quinine intake, dubbed Q*ui *(quinine sensitivity, taste), in a close proximity with the *Prp2 and Prh1 genes *on distal Chr 6 [139,151,152]. Lush also reported linkage to this region for aversion to other bitter compounds, including cycloheximide, raffinose undecaacetate, and copper glycinate (Figure 4; [136,151,153]) and sagely hypothesized the existence of a cluster of bitter taste genes at this location, which "may have evolved from one original bitterness gene by a process of local duplication and differentiation" [136]. A recently characterized bitter taste receptor gene family in mice (36 *Tas2r *genes) forms two clusters on distal Chr 6 and one on Chr 15, including 24 genes at the *Soa *and *Qui *loci that collectively comprise a large haplotype for quinine taste sensitivity (Figure 5) [30,111,113,114]. Indeed, analysis suggests extensive gene duplication among the *Tas2r *genes [114]. Physical mapping of the *Soa-Qui *locus in strains of quinine taster (B6) and "non-taster" (DBA/2J) mice reveals considerable variance across the *Tas2r *genes [30]: Only two of 24 alleles were identical in both strains at the amino acid level. Furthermore, several of genes were either pseudogenes or deleted altogether in the nontaster strain, although a specific receptor-ligand relationship has not yet been uncovered.

Among bitter receptor genes in the mouse, only two *Tas2r*s have been de-orphanized thus far with respect to ligand, one responding exclusively to cycloheximide, the other to PROP and denatonium [5,154,155]. Several human T2R receptors, including *TAS2R38*, have also been characterized with respect to ligand binding (Table 2).

### Evolution of the bitter taste receptor gene family

Prominent features of the bitter receptor gene family in vertebrates include a high rate of non-synonymous (i.e. resulting in amino acid change) substitutions, and a relatively conserved family size (~15 – 33 functional genes) with multiple orthologues among mammalian species [156]. The most divergent portions of the *Tas2r *genes are those that code for extracellular domains, a possible site of ligand binding [114,157]. The particular nature of the high rate of mutations in this gene family has led to speculation of relaxation of selective constraint and loss of function in primate species [158,159]. On the other hand, analysis of *TAS2R *haplotypes among humans shows a greater diversity than expected, and suggests strong effects of natural selection [6,160]. The number of functional receptors may have decreased in humans and other primates as the chemosensory function of these species has diminished in importance relative to other mammals, but positive selection has shaped the evolution of bitter taste receptors in response to variation in local plant species that are toxic.

## Salt taste

A prototypical salty taste stimulus is NaCl, but several other compounds (e.g., chlorides and other salts of Na, Li and K) also evoke salty taste. Sodium enters epithelial cells through the sodium-selective epithelial channel ENaC. ENaC is a member of the degenerin/ENaC superfamily of ion channels and is inhibited by a diuretic, amiloride. Taste receptor cells express amiloride-blockable, Na^+^-selective channels that share a number of physiological properties with the ENaCs (for review, see [161]). Thus, a large portion of NaCl taste is likely dependent on the influx of sodium through ENaC. However, whether ENaC plays the role of a salt taste receptor requires further studies.

Sodium is an important nutrient, and its consumption is under a strong homeostatic control [162,163]. Therefore, results of the long-term preference tests with sodium salts may be affected not only by perception of their taste, but also by postingestive effects. Thus, additional evidence is usually needed to link variation in salt consumption with differences in salty taste perception.

Salt consumption may be under genetic control in humans [164]. Inbred strain differences in voluntary NaCl consumption have been reported in both mice [28,66,107,166-169] and rats [170-174]. Although these genetic differences in NaCl intake or preference are often profound, it is not clear whether they originate from differences in taste perception or are due to other factors. A few studies undertook a genetic analyses of NaCl consumption in mice [73] and rats [175-179]. In rats, salt intake was found to be linked to the Y Chr [177,180]. No linkage data for NaCl consumption in mice have been published.

Despite large strain variation in NaCl preferences, most studies found either small or no differences in integrated responses of the gustatory nerves to NaCl among strains of mice [108,166,181-183] and rats [184,185], with some exceptions [186].

In rodents, lingual application of amiloride suppresses the chorda tympani responses to NaCl [187]. There are mouse and rat strain differences in sensitivity of the chorda tympani response to amiloride. Initial studies suggested that amiloride sensitivity of the CT nerve response to NaCl is associated with behavioral responses to NaCl: strains with stronger amiloride-induced suppression of the neural response to NaCl had stronger NaCl avoidance [166,182,183,186]. However, this relationship was not found in all studies. For example, NZB/BlNJ and CBA/J mice dramatically differ in NaCl consumption [107,168] but have chorda tympani responses to NaCl of similar magnitude and sensitivity to amiloride [181]. Lack of correspondence between amiloride sensitivity of the CT response to NaCl and NaCl consumption was also found when three strains of rats, Fisher 344, Wistar and Sprague-Dawley, were compared [185,186]. Furthermore, amiloride was found to affect NaCl perception in mice regardless of amiloride sensitivity of their gustatory neural responses to NaCl [32,188]. More studies are needed to determine whether there is a mechanistic relationship between amiloride sensitivity of neural responses to NaCl and NaCl preference. It appears that the genetic variation in voluntary NaCl consumption can depend on a variety of factors, including, but not limited to, peripheral taste responsiveness.

Rats typically prefer NaCl solutions of intermediate (osmotically hypotonic or isotonic) concentrations. However, rats from the Fisher 344 strain do not display NaCl preferences [170]. A series of subsequent studies have presented a detailed analysis of mechanisms underlying this lack of NaCl preference in Fisher 344 rats. Relative to rats with typical responses to NaCl, Fisher 344 rats develop attenuated salt appetite in response to sodium deficiency or to interference with renin-angiotensin-aldosterone system, but they have similar sodium loss during sodium depletion. This suggests that differences in hedonic perception of NaCl rather than differences in sodium metabolism underlie aberrant NaCl consumption by the Fisher 344 rats [189-191]. Consistent with this, Fisher 344 rats show decreased appetitive and increased aversive oral motor responses to NaCl [192], and their NaCl aversion is abolished by CT, but not IX nerve transection [193,194]. However, NaCl detection thresholds were not altered in Fisher 344 rats [195]. Thus, it appears that NaCl aversion in Fisher 344 rats is mediated by aversive gustatory information conveyed by the CT nerve.

Increased NaCl consumption by SHR (spontaneously hypertensive) rats does not depend on strain differences in peripheral gustatory input [184,196], but appears to be mediated by the brain renin-angiotensin system [197,198].

## Taste and other complex phenotypes

Taste perception involves hedonic processes and is an important factor affecting ingestive behavior. Therefore, genetic variation in taste preferences is likely to affect complex phenotypes that depend on oral consumption of nutrients or drugs, or involve pleasure-seeking behavior. There are several examples of association of taste and complex traits in humans, including relationship between sweet taste and obesity [34,41,199,200] or alcohol intake [201-205], and between bitter taste and alcohol intake [206-212], smoking [213,214], food choice [215] and other health-related traits [216]. Studies of rodents elucidated some genetic factors and physiological mechanisms for association between sweet taste and alcohol, and have potential for unveiling mechanisms of the other associations between taste and complex phenotypes.

The oral consumption of alcohol is accompanied by chemosensory perception of its flavor, which plays an important role in its acceptance and rejection. Three independent sensory systems – taste, olfaction and chemosensory irritation – are involved in the perception of alcohol flavor. Humans perceive alcohol as a combination of sweet and bitter tastes, odors and oral irritation (e.g., burning sensation), all of which vary as a function of concentration [209,217,218]. Likewise, rodents detect the sweet (sucrose-like) and bitter (quinine-like) taste [219-221], odor volatiles [222,223] of alcohol, and probably the other components detected by humans [224,225].

Perception of the sweet taste component of ethanol by rodents was shown in behavioral and neurophysiological experiments. CTAs generalize between ethanol and sucrose [219,221,223,226,227]. Electrophysiological recordings indicate that lingual application of ethanol activates sweet-best neural fibers in the gustatory nerves [228,229] and sweet-best units in the nucleus of the tractus solitarius [230,231]; this activity is blocked by application of gurmarin, a peripheral antagonist of sweet taste [231]. Central mechanisms that determine hedonic responses to ethanol and sweeteners also overlap and involve opioidergic, serotonergic and dopaminergic brain neurotransmitter systems [232-236]. In addition, there may be common signals related to the caloric value of ethanol and sugars [237-242].

Positive correlations between preferences for ethanol and sweeteners in rats and mice were found among various strains and in segregating crosses [25,45,52,66,72,73,243-246], reviewed in [62,63]. This genetically determined association can be underlaid by any of the mechanisms described above, including peripheral or central taste processing, or postingestive reward.

Genetic analysis of a cross between mice from a high ethanol- and sweetener-preferring B6 strain and a low ethanol- and sweetener-preferring 129 strain suggested that the strain differences in sweetener and ethanol consumption depend on relatively small and partially overlapping sets of genes [73]. One of these genetic loci, *Ap3q *(alcohol preference 3 QTL), maps to the subtelomeric region of Chr 4 [93] overlapping with the saccharin preference (*Sac*) locus that corresponds to the sweet taste receptor gene, *Tas1r3 *[77]. This suggests that the *Tas1r3 *gene is identical to the *Ap3q *locus and that its pleiotropic effect on ethanol consumption is mediated by genetic differences in perception of the sweet taste component of ethanol flavor: higher hedonic attractiveness of ethanol sweetness results in higher ethanol intake by B6 mice. The role of the T1R3 receptor in alcohol consumption was confirmed in a recent study showing that mutant mice lacking the *Tas1r3 *gene have diminished ethanol intakes and preferences [247]. In addition to the *Tas1r3 *gene, there are other genetic loci with pleiotropic effects on ethanol and sweetener intake [248,249].

## Conclusion

Behavioral genetic studies of taste responsiveness have been instrumental in the discovery of G-protein-coupled taste receptors. Genetic mapping of a human PTC/PROP taste sensitivity locus and mouse bitter and sweet taste loci have facilitated identification of the T1R and T2R families of taste receptors. The behavioral genetic approach has potential for more discoveries of taste mechanisms. There is strong evidence that multiple genes control behavioral taste responses. These yet unknown genes are likely to be involved in different stages of the taste processing pathway, including taste reception, transduction and transmission in the periphery and in the brain, and interaction of taste processing with homeostatic systems involved in the regulation of feeding, appetite, fluid balance, and reward. Genetics has experienced dramatic progress in recent years, with genome sequencing completed for several species, including mouse and human. These advances in genomic resources tremendously facilitate chromosomal mapping of genes affecting taste responsiveness and their identification. This turns behavioral genetics into a powerful approach for understanding mechanisms of taste.

## Competing interests

The authors declare that they have no competing interests.

## Authors' contributions

JDB and AAB contributed equally to this review.
